# Di-4-ANEPPS Modulates Electrical Activity and Progress of Myocardial Ischemia in Rabbit Isolated Heart

**DOI:** 10.3389/fphys.2021.667065

**Published:** 2021-06-10

**Authors:** Marina Ronzhina, Tibor Stracina, Lubica Lacinova, Katarina Ondacova, Michaela Pavlovicova, Lucie Marsanova, Radovan Smisek, Oto Janousek, Katerina Fialova, Jana Kolarova, Marie Novakova, Ivo Provaznik

**Affiliations:** ^1^Department of Biomedical Engineering, Faculty of Electrical Engineering and Communication, Brno University of Technology, Brno, Czechia; ^2^Department of Physiology, Faculty of Medicine, Masaryk University, Brno, Czechia; ^3^Centre of Biosciences, Institute of Molecular Physiology and Genetics, Slovak Academy of Sciences, Bratislava, Slovakia; ^4^International Clinical Research Center, St. Anne’s University Hospital Brno, Brno, Czechia

**Keywords:** di-4-ANEPPS, rabbit isolated heart, myocardial ischemia, electrogram analysis, patch-clamp, voltage-sensitive dye

## Abstract

**Aims:**

Although voltage-sensitive dye di-4-ANEPPS is a common tool for mapping cardiac electrical activity, reported effects on electrophysiological parameters are rather. The main goals of the study were to reveal effects of the dye on rabbit isolated heart and to verify, whether rabbit isolated heart stained with di-4-ANEPPS is a suitable tool for myocardial ischemia investigation.

**Methods and Results:**

Study involved experiments on stained (*n* = 9) and non-stained (*n* = 11) Langendorff perfused rabbit isolated hearts. Electrophysiological effects of the dye were evaluated by analysis of various electrogram (EG) parameters using common paired and unpaired statistical tests. It was shown that staining the hearts with di-4-ANEPPS leads to only short-term sporadic prolongation of impulse conduction through atria and atrioventricular node. On the other hand, significant irreversible slowing of heart rate and ventricular conduction were found in stained hearts as compared to controls. In patch clamp experiments, significant inhibition of sodium current density was observed in differentiated NG108-15 cells stained by the dye. Although no significant differences in mean number of ventricular premature beats were found between the stained and the non-stained hearts in ischemia as well as in reperfusion, all abovementioned results indicate increased arrhythmogenicity. In isolated hearts during ischemia, prominent ischemic patterns appeared in the stained hearts with 3–4 min delay as compared to the non-stained ones. Moreover, the ischemic changes did not achieve the same magnitude as in controls even after 10 min of ischemia. It resulted in poor performance of ischemia detection by proposed EG parameters, as was quantified by receiver operating characteristics analysis.

**Conclusion:**

Our results demonstrate significant direct irreversible effect of di-4-ANEPPS on spontaneous heart rate and ventricular impulse conduction in rabbit isolated heart model. Particularly, this should be considered when di-4-ANEPPS is used in ischemia studies in rabbit. Delayed attenuated response of such hearts to ischemia might lead to misinterpretation of obtained results.

## Introduction

Animal models are widespread tool for studying cardiac electric activity under various experimental conditions. Electric activity of the myocardium is often monitored as cardiac action potential (AP). AP can be recorded directly by microelectrodes or using optical methods. The conventional methods using microelectrodes are the gold standards for measuring electrical signals on the cellular level (Richardson and Xiao, 2010). However, these methods require an electrode placement in tight contact to the cell surface, which is difficult to ensure in long-term experiments. Optical methods use fluorescence properties of special chemical compounds, so called voltage-sensitive dyes (VSD). Fluorescence of VSD molecules bound on the cell membrane is proportional to transmembrane potential. Briefly, the procedure of AP measurement using VSD consists of: (a) loading the heart with the dye for a definite time; (b) washing the heart with perfusion solution to remove the unbound molecules; (c) exposing the heart to excitation light (with halogen lamp, xenon/mercury arc lamp, LED or laser); (d) detecting the emission light with some photodetector (mostly photodiodes, photodiode array, photomultiplier tubes, and CCD cameras) (Salama, 2001; Efimov et al., 2004; Dhein et al., 2005; Ronzhina et al., 2013a; O’Shea et al., 2020). Various VSDs with different properties have been introduced and are commercially available (Lopez-Izquierdo et al., 2014; Acker et al., 2020). The most commonly used VSDs are RH-237 and di-4-ANEPPS. Di-4-ANEPPS allows reaching a time resolution better than 1 ms (Dhein et al., 2005) and exhibits changes in fluorescence of up to 10% per 100 mV (Johnson et al., 1999). VSD based approach allows non-invasive record of AP from a larger area of heart surface with high spatial resolution.

Despite such benefits, the application scale of optical methods is generally limited because of the properties of available fluorescent dyes (Ronzhina et al., 2013a). One of the most important disadvantages of VSDs are their possible side effects on cardiac electrophysiology. Many authors reported various effects of di-4-ANEPPS in various experimental models, including: (a) increasing contractility of cardiac muscle in rabbit isolated heart and human atrial preparation (Cheng et al., 1998); (b) AP duration prolongation in guinea pig isolated ventricle myocytes (Hardy et al., 2006) and in left ventricle mid-myocardial myocytes of beagle dog (Hardy et al., 2009); (c) QRS duration prolongation (Larsen et al., 2010), decrease of heart rate (Fialova et al., 2010) and slowing cardiac impulse propagation (Larsen et al., 2012) in guinea pig isolated heart; (d) PQ interval prolongation, transient blocks of atrioventricular (AV) conduction, decrease of perfusion pressure due to dilation of coronary arteries (Nygren et al., 2003), decrease of heart rate and slight prolongation of QRS and QTc duration (Fialova et al., 2011) in rat isolated heart; (e) vasoconstriction (Nygren et al., 2000), increase of total activation time (Liang et al., 2006), and atrioventricular block and transient polymorphic ventricular tachycardia (Salerno et al., 2020) in mouse isolated heart. Some above mentioned effects are dose dependent (Larsen et al., 2012) and some seem to be caused by phototoxic effect of the dye (Hardy et al., 2009) or by other mechanisms associated with the binding of the dye to the cardiac cell membrane (Hardy et al., 2006). The most of above listed observations are the secondary outcomes obtained during experiments and there are only a few studies primarily focused on the evaluation of di-4-ANEPPS undesirable effects.

Assuming the abovementioned, it is evident that the response of myocardium to staining with di-4-ANEPPS has been studied mainly in small rodents such as mouse, rat and guinea pig. Nevertheless, it is known that the rabbit model is more suitable for cardiovascular studies due to the high similarity to human in cardiac electrophysiology parameters (Kaese et al., 2013), distribution of ion channels, time course of repolarization, and calcium handling (Bers, 2002). Rabbit isolated heart perfused according to Langendorff is frequently used for research of myocardial ischemia, infarction, and ischemia-related arrhythmias. The electrical activity in such studies is evaluated by conventional and/or optical methods (Mandapati et al., 1998; Qian et al., 2003; Dhein et al., 2005; Holcomb et al., 2009; Matiukas et al., 2009; Brack et al., 2013; Kang et al., 2016). However, the eventual side effects of VSDs in optical mapping studies are only briefly discussed or not addressed at all.

Based on our preliminary observations in other species (Novakova et al., 2008; Fialova et al., 2010; Vesely et al., 2015), existence of electrophysiological effects of di-4-ANEPPS on rabbit myocardium can be expected. Therefore, this study is focused on the description of the effect(s) of di-4-ANEPPS on rabbit isolated heart. Advanced electrogram analysis was performed during the staining with the dye, its washout as well as during ischemia-reperfusion. Moreover, the effect of di-4-ANEPPS on sodium current (I_Na_) was evaluated in NG108-15 cells. The goal of the study was to improve the reproducibility of the results from the studies using di-4-ANEPPS.

## Materials and Methods

The animal experiments were conducted according to the recommendations of the European Community Guide for the Care and Use of Laboratory Animals. The experimental protocol was approved by the Committee for Ensuring the Welfare of Experimental Animals, Faculty of Medicine, Masaryk University.

The experiments were performed on the New Zealand rabbits of both sexes (*n* = 20, average body weight 2.65 ± 0.67 kg, Velaz, s.r.o., Czech Republic).

The VSD di-4-amino-naphthyl-ethenylparidiunium (di-4-ANEPPS, Molecular Probes, Inc., United States) was dissolved in dimethyl sulfoxide (DMSO, Sigma-Aldrich, United States) in the concentration of 2 mM (stock solution) and stored in refrigerator (at 4°C). For staining, the di-4-ANEPPS stock solution was diluted either in the Krebs-Henseleit (K-H) perfusion solution (for isolated rabbit heart) or in bath solution (for NG108-15 cells) to the final concentration of 2 μM. The K-H solution contained (in mM): 118 NaCl, 24 NaHCO_3_, 4.2 KCl, 1.2 KH_2_PO_4_, 1.2 MgCl_2_, 1.2 CaCl_2_, 5.5 glucose, and 10 taurine.

### Langendorff-Perfused Heart Experiments

#### Experimental Setup

The animals were randomly divided into two experimental groups: stained (*n* = 9) and control (*n* = 11). The animals were deeply anesthetized by i.m. application of xylazine (2 mg/kg) and ketamine (60 mg/kg). The trachea was cannulated and the animal was artificially ventilated to avoid peri-preparational ischemia. The chest was opened, the heart was excised and placed into a cold (5°C) K-H solution. The aorta was prepared and cannulated, the heart was then placed into the bath filled with K-H solution and perfused with the K-H solution aerated by gas mixture (95% O_2_ and 5% CO_2_) at stable conditions (pressure of 80 mmHg and temperature of 37°C). The heart was allowed to stabilize for 20 min.

**FIGURE 1:**
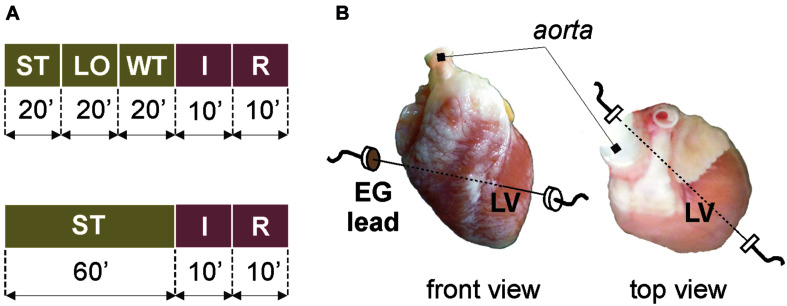
Isolated heart experiments: **(A)** protocol of experiments with (*top*) and without (*bottom*) voltage-sensitive dye di-4-ANEPPS; **(B)** placement of recording electrodes. ST, stabilization; LO, loading with the dye; WT, washout; I, ischemia; R, reperfusion; EG, electrogram; LV, left ventricle.

Different experimental protocols were applied in stained and non-stained (control) groups (see Figure 1A). After stabilization period in the stained group (Figure 1A, top), the heart was loaded with the di-4-ANEPPS for 20 min. Such slow staining by perfusion with low-concentrated dye allows recording AP data for 1–2 h without losing their quality, which is crucial in case of long-term experiments (e.g., experiments with repeated ischemia) (Novakova et al., 2008). Then, the dye was washed out for 20 min. The washout period was followed by global ischemia and reperfusion (10 min each). The protocol for control group (Figure 1A, bottom) did not include the VSD loading and washout; therefore, the stabilization period was prolonged to 60 min to the same protocol duration before ischemia onset.

To monitor the overall stability of the preparation during the experiment, coronary flow was regularly measured as the outflow of the solution from the bath. During the whole experiment, electrogram (EG) was recorded by touch-less method using the bipolar lead of Ag–AgCl disk electrodes embedded into the bath (see Figure 1B). This set-up allows to record data without movement artifact from the middle of the left ventricle, where the prominent morphological patterns are present in EG under non-ischemic as well as ischemic conditions (Ronzhina et al., 2013b). To ensure appropriate quality of data, EG was sampled with 2 kHz rate at 16-bit resolution.

#### Electrogram Analysis and Arrhythmia Evaluation

The parts of EGs distorted with artifacts were not included into the analysis. The low-frequency baseline wander was eliminated and QRS complexes were detected with previously designed algorithm (Hejc et al., 2015). The onset of P wave and QRS complex, J point and the T wave offset were detected through the whole records.

Delineation results were used to calculate parameters representing rhythm and morphology of analyzed EG: (a) duration of P wave (P_D_), PQ interval (PQ_D_), QRS complex (QRS_D_), RR (RR_D_) and QT (QT_D_) intervals, and QT intervals corrected using Bazett’s formula (QT_C_); (b) absolute value of maximal QRS deviation (QRS_A_) and maximal deviation of ST-T interval (T_A_). Particularly, P_D_, PQ_D_, and QRS_D_ were calculated to assess the conduction through atria and ventricles, respectively. RR_D_ represented sinoatrial (SA) node activity. QT_D_ and its corrected value QT_C_ were used to detect possible arrhythmogenic effect of the dye. QRS_A_ and T_A_ were previously considered as the most suitable for early ischemia assessment in the present animal model and recording setup (Ronzhina, 2017; Ronzhina et al., 2017). Cardiac electrical activity under variable experimental conditions was represented by averaged values of parameters calculated in each minute of the experiment.

The parameters in ischemia and reperfusion were additionally corrected by subtracting the corresponding value derived immediately before ischemia onset (i.e., the value in the 60th minute of experimental protocol, which corresponds to the end of washout or the end of stabilization in experiments with or without dye, respectively). Corrected parameters are denoted by Δ (e.g., ΔRR_D_ and ΔQRS_D_, etc.).

Additionally, normal sinus rhythm (SR), junctional rhythm (NOD) and atrioventricular block (AVB) of the 2nd and the 3rd degree were evaluated manually through the whole data set. Ventricular premature beats (VPBs) and supraventricular extrasystoles (SVES) were counted within particular experimental periods.

### Cell Culture Experiments

#### Cell Cultivation

NG108-15 cells were cultivated in Dulbecco’s Modified Eagle Medium (DMEM, Sigma Aldrich, Slovakia) supplemented with heat-inactivated calf fetal serum (15%), Penicillin/Streptomycin (1%), Pyruvic acid (1%), and Plasmocine (0.2%) at 37°C in a humidified 5% CO_2_, 20% O_2_ and 75% N_2_ atmosphere. For experiment, the cells were seeded on a PLL-coated coverslips in the density of 3 × 10^4^ cells per a 12 mm coverslip. Addition of 1 mM N6,2′-*O*-Dibutyryladenosine 3′,5′-cyclic monophosphate (dbcAMP) evoked differentiation of NG108-15 cells into neuron-like cells and increased expression of voltage-gated ion channels. Experiments were performed 5–7 days after the plating.

#### Whole-Cell Patch Clamp Experiments

Sodium current I_Na_ was measured in the whole-cell patch clamp configuration using the HEKA EPC10 amplifier (HEKA Electronics, Lambrecht, Germany). The extracellular solution contained (in mM): 115 NaCl, 10 BaCl_2_, 3 CsCl, 10 HEPES, and 0.5 MgCl_2_ at a pH 7.4 (with NaOH). Intracellular solution contained (in mM): 135 CsCl; 3 Na_2_-ATP; 20 TEA-Cl; 3 EGTA; 10 HEPES; 0.4 Na_2_-GTP; and 2 MgCl_2_ at a pH 7.4 (with CsOH). Osmolarity of the solutions was measured by Osmomat 030-Gonotec (Germany). Osmolarity of the intracellular solution reached approximately 290–300 mOsmol/l. Osmolarity of the extracellular solution was by 2–3 mOsm/l lower as compared to the intracellular one. The adjustment of the osmolarity of extracellular solution was done by adding glucose.

The cells (*n* = 31) were incubated for 30 min in the dye solution (slow application procedure similar to that used in isolated heart experiments) and current–voltage (I–V) relations for sodium current were recorded by a series of 5 ms long depolarizing voltage pulses to the voltages ranging from −70 to +70 mV applied from a holding membrane potential of −90 mV every 5 s. The same recording series was performed in non-stained cell group (*n* = 27). Additionally, the experimental procedure was repeated (*n* = 19) using dye-free DMSO (of the same concentration) and control group (*n* = 16) to reveal the potential effects of the solvent itself.

Data were recorded by HEKA Patchmaster v2x73.3 (HEKA Electronics; Lambrecht/Pfalz, Germany) and analyzed by HEKA Fitmaster v2x73.3 and Origin 8.1 (OriginLab Co., Northampton, MA, United States). Whole-cell current densities were derived as peak current amplitude divided by corresponding cell capacitance.

### Statistical Analysis

Statistical analysis was carried out for data recorded in isolated hearts during two main parts of experimental protocols separately–before and after ischemia onset, respectively (green and red part of block flows from Figure 1A, respectively). The normality and homoscedasticity of the parameters were checked with Shapiro-Wilk test and Levene’s test, respectively. It was confirmed that both assumptions are not precisely hold. Non-parametric statistical tests were therefore performed to compare different data. Particularly, Wilcoxon sign rank paired test was used to compare EG parameters calculated in stained or non-stained hearts at different time moments of the experiment. Unpaired Mann-Whitney *U*-test was performed to compare EG parameters and number of VPBs calculated in stained hearts with those from the non-stained hearts.

Areas under receiver operating characteristic curves (AUCROCs) were calculated from EG parameters to assess their ability to detect myocardial ischemia. For more detailed analysis, AUCROCs were separately derived for every minute of ischemia. AUCROC value of 0.5 indicates random (i.e., useless) discriminating, while value of 1 indicates perfect discrimination performance of the parameter.

Results of patch clamp experiments are presented as a mean ± standard error of mean (SEM). The differences between the groups were analyzed by one-way analysis of variance (ANOVA) and Tukey post-hoc test.

For all above tests, *p*-value less than 0.05 was considered statistically significant.

## Results

According to the coronary flow, heart rate and cardiac rhythm, all the isolated hearts included in the study were considered stable at the end of the stabilization period. During the VSD loading in the stained group, the mean coronary flow was decreased by 14% (compared to the end of the stabilization). The washout did not affect the mean coronary flow which remained constant until the onset of the ischemia. The mean coronary flow in the control group was stable until the onset of the ischemia.

### Data Analysis Under Non-ischemia Conditions

#### Morphological Parameters of Electrogram

**FIGURE 2:**
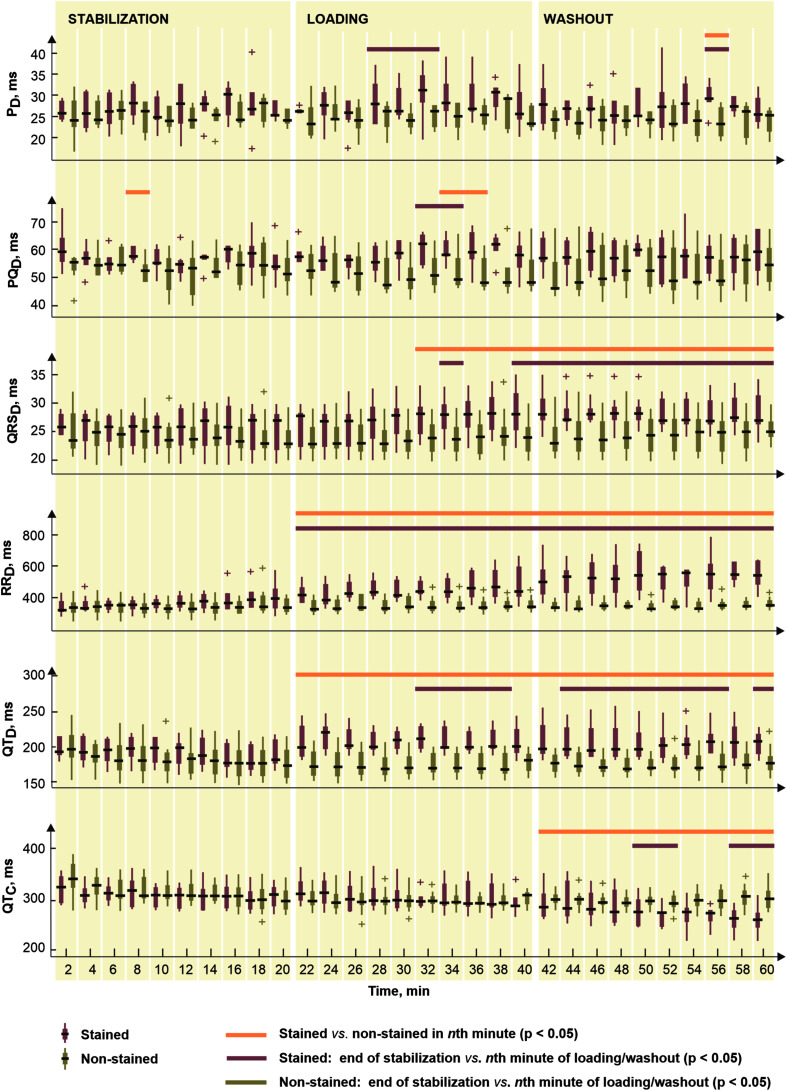
Distributions of electrogram rhythm parameters in stained (*n* = 9) and non-stained (*n* = 11) hearts: P_D_, PQ_D_, QRS_D_, RR_D_, QT_D_, and QT_C_, duration of P wave, PQ interval, QRS complex, RR and QT intervals, and corrected QT intervals, respectively. Displayed for every second minute of the period as median, the edges of the box indicate 25th–75th percentiles. Stabilization, dye loading and washout correspond with the notation used in experimental protocol with di-4-ANEPPS. Horizontal lines indicate significant differences (*p* < 0.05) revealed via paired and unpaired tests. “+” indicates outlier.

**FIGURE 3:**
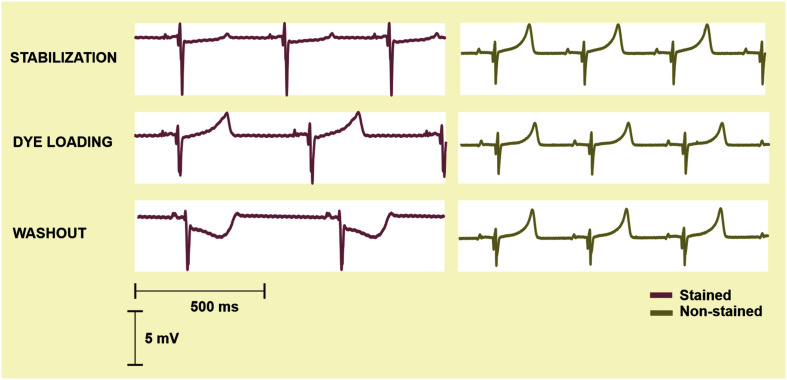
Representative electrograms recorded in the stained rabbit isolated heart (*left*) at the end of stabilization (*top*), loading with di-4-ANEPPS (*middle*) and washout (*bottom*) and in the non-stained rabbit isolated heart (*right*) in corresponding minutes of the experiment. Prolongation of RR, QT, and QRS appears during the loading procedure and remains obvious till the end of washout in the stained heart.

To reveal potential direct effects of di-4-ANEPPS on electric activity of the heart various EG parameters were evaluated before the ischemia onset (green part of block flows from Figure 1A). Distributions of EG parameters in corresponding time interval are shown in Figure 2. Generally, data from stained hearts are more variable than those from non-stained ones in terms of alteration in time as well as inter-subject variability at the particular time points (see median values and interquartile ranges in boxplots from Figure 2, respectively). According to the results of paired test, EG parameters computed in loading and washout periods in stained hearts vary from those calculated at the end of stabilization (see Figure 2). Significantly increased (transient in case of P_D_ and PQ_D_ and sustained in case of QRS_D_, RR_D_, and QT_D_) values were observed in this group from approx. the 4th–12th minute of dye loading. Particularly, median values of QRS_D_, RR_D_, and QT_D_ at the end of washout were approx. by 11, 22, and 19% higher than those derived at the end of stabilization (also illustrated on raw EGs in Figure 3). On the contrary, decreased QT_C_ values (approx. 22% decrease of median value) were found in stained hearts from the loading till the end of washout. However, it is likely due to RR_D_, which is changing continually from the beginning of loading period, whereas QT_D_ is quite stable except of sudden prolongation in the first minute of dye loading response to prolonged RR.

In non-stained hearts, no significant changes in EG parameters were found through the stabilization, as compared to the values from the 20th minute of stabilization.

Above results correspond to the results of unpaired test (see Figure 2). In case of P_D_ and PQ_D_, significant differences between stained and non-stained groups were only sporadically observed in washout and dye loading, respectively. On the contrary, QRS_D_, RR_D_, and QT_D_ differed significantly between the groups from the beginning or the middle of loading till the end of washout. Particularly, in the 60th minute of experiment, median values of QRS_D_, RR_D_, and QT_D_ in stained hearts were approx. by 15, 37, and 20% higher, respectively, and median value of QT_C_ in the same group was approx. by 17% lower as compared to the non-stained hearts.

Among morphological parameters, only sporadic changes appeared in QRS_A_ and T_A_ during dye loading and washout in the stained hearts, as compared to the end of stabilization. In non-stained hearts, the parameters remained stable through the whole 1-h stabilization (see paired test results in Figure 2).

#### Arrhythmia Incidence

**FIGURE 4:**
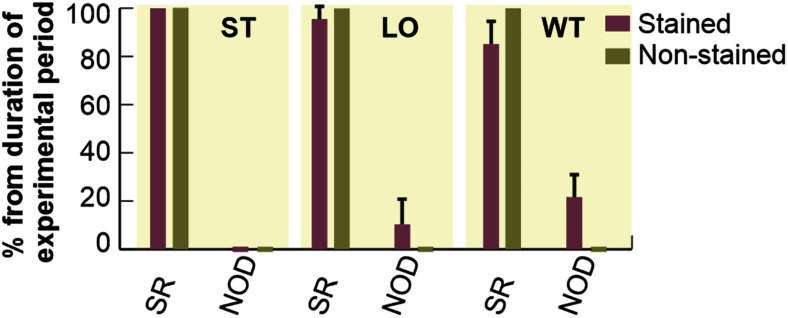
Incidence of most frequently observed types of cardiac rhythm in electrograms. Expressed as a percentage of total duration of corresponding experimental period and shown as a bar with the top edge indicating mean value for the group and a whisker indicating SEM. SR, normal sinus rhythm; NOD, junctional rhythm; ST, LO, and WT, stabilization, dye loading and washout, respectively (according to notation used in experimental protocol with di-4-ANEPPS).

Duration of different types of cardiac rhythm expressed as percentage of the total duration of particular experimental period is shown in Figure 4. In the first 20 min of experiment (i.e., stabilization), no pathological episode was found in both groups. In stained hearts, only short-term episodes of NOD (total duration of about 5%) were detected during loading. On the contrary, this type of arrhythmia was quite frequent (about 16%) in washout.

Atrioventricular block and SVES were only sporadically present in both experimental groups with total of 0–4 episodes per experiment. VPBs were sporadically present in 36% (*n* = 4, up to 2 episodes) of the non-stained hearts during the 60-min stabilization period. In stained hearts, sporadic VPBs (2 episodes on average) were presented in 33% (*n* = 3), 67% (*n* = 6) and none of the hearts during stabilization, dye loading and washout, respectively.

#### Sodium Current in Differentiated NG108-15 Cells

**FIGURE 5:**
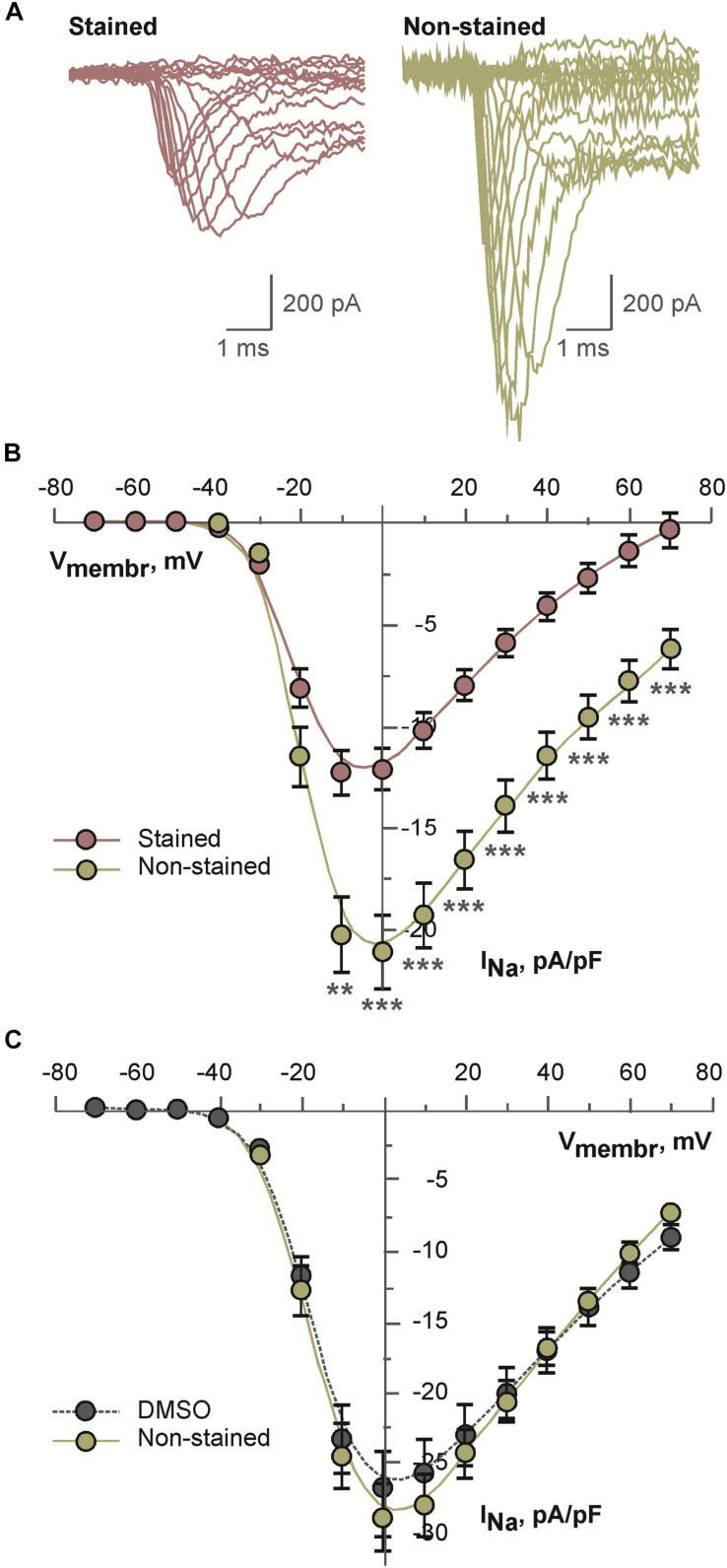
Results of patch-clamp analysis of sodium channels in differentiated NG108-15 cells: **(A)** representative examples of current traces recorded after 30 min long incubation of cells in the presence of 2 μM di-4-ANEPPS (stained) or under the control conditions (non-stained); **(B)** averaged current-voltage relations for sodium current recorded after 30 min long incubation of cells in the presence of 2 μM di-4-ANEPPS (stained, *n* = 31) or under the control conditions (non-stained, *n* = 27); **p < 0.01; ***p < 0.001 (ANOVA and Tukey test); **(C)** averaged current-voltage relations for sodium current recorded after 30 min long incubation of cells in the presence of dye-free DMSO in the dilution 1:1,000 (DMSO, *n* = 19) or under the control conditions (non-stained, *n* = 16). In panels **(B,C)**, solid lines represent B-spline fit to the experimental data.

Above mentioned effects may be explained by altered voltage-dependent ion channels. Therefore, possible effects of the dye on voltage-dependent sodium current were tested. As a model for these experiments differentiated NG108-15 cell were used. This cell line expresses multiple isoforms of sodium channels. Representative examples of sodium current traces recorded from the cells incubated in the presence of 2 μM di-4-ANEPPS or under the control conditions are shown in the Figure 5A. Current density of sodium current measured in the stained cells was consistently and significantly inhibited in the whole range of membrane voltages as compared to the non-stained cells (see Figure 5B). To exclude an effect of the solvent itself cells were incubated for the same time in DMSO. Under these conditions, sodium current density was not altered (Figure 5C).

### Electrogram Analysis Under Ischemic Conditions

According to the above results, loading of the rabbit isolated heart with di-4-ANEPPS leads to the irreversible changes of its electrical activity, which is manifested in significantly increased or decreased values of some EG parameters. To test the hypothesis that above effects may have an impact on progress and evaluating the response of previously stained rabbit myocardium to ischemia, EGs recorded in the second part of experimental protocol (i.e., during ischemia and reperfusion) were analyzed separately.

#### Arrhythmia Incidence

**FIGURE 6:**
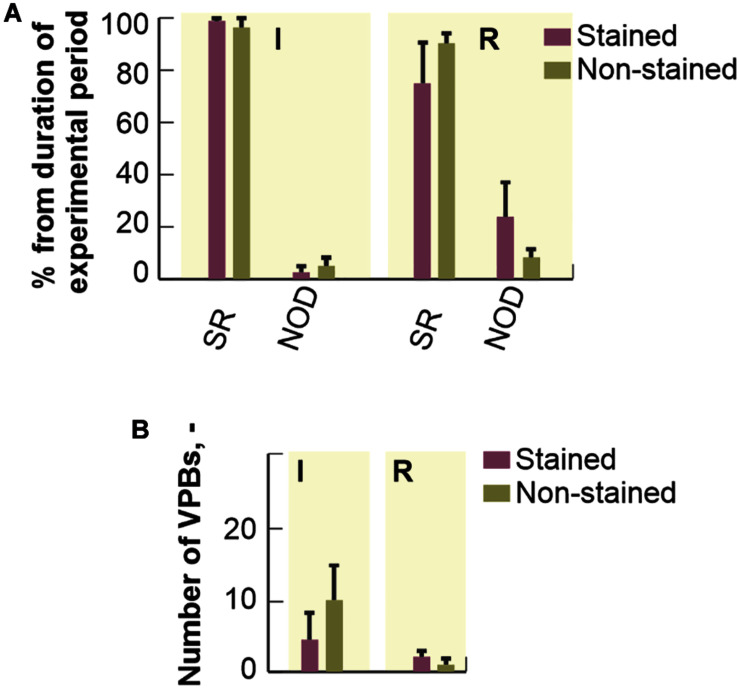
Distribution of different cardiac rhythms throughout ischemia and reperfusion: expressed as a percentage of total duration of corresponding experimental period and shown as a bar with the top edge indicating mean value for the group and a whisker indicating SEM. **(A)** The distribution of a number of ventricular premature beats (VPBs): shown as a bar with the top edge indicating mean number of VPBs in the group and a whisker indicating SEM **(B)**. SR, normal sinus rhythm; NOD, junctional rhythm; I, ischemia; R, reperfusion.

Percentage duration of SR and NOD related to the total duration of ischemic and reperfusion periods is shown in Figure 6A. Arrhythmia episodes were more frequent in the stained hearts as compared to the non-stained ones, where in approx. half of experiments normal sinus rhythm was detected throughout the whole period. In the stained hearts, NOD was mainly present in the reperfusion period (up to 25% of reperfusion and up to 3% of ischemia, respectively). In both groups, AVBs were only sporadically presented.

No significant differences (Mann-Whitney *U*-test, *p* < 0.05) in mean number of VPBs (see Figure 6B) were found between the stained and the non-stained hearts in ischemia as well as in reperfusion. VPBs were present in 67% (*n* = 6) of the stained hearts and in 64% (*n* = 7) of the non-stained ones. In both groups, about 95% of all VPBs occurred in the middle of ischemia.

#### Morphological Parameters of Electrogram

**FIGURE 7:**
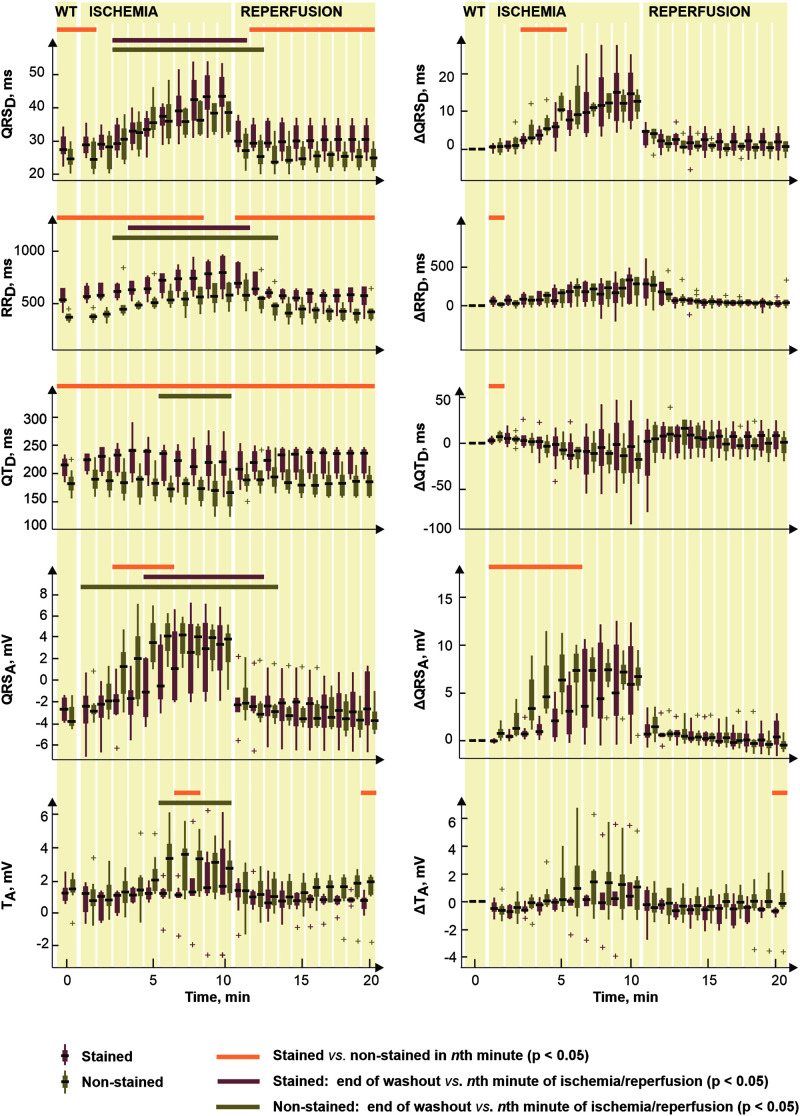
Distributions of selected electrogram parameters (*left*) and corresponding corrected values Δ calculated by subtracting end-washout value from the current one (*right*). Shown for stained (*n* = 9) and non-stained (*n* = 11) hearts. QRS_D_, RR_D_, QT_D_, QRS_A_, and T_A_, duration of QRS complex, RR and QT intervals and maximal absolute QRS deviation and maximal deviation of ST-T interval, respectively. Displayed for end-washout (WT, according to the notation used in experimental protocol with di-4-ANEPPS) minute and every minute of ischemia and reperfusion as median; the edges of the box indicate 25th–75th percentiles. Horizontal lines indicate significant difference (*p* < 0.05) revealed via paired (not calculated for Δ values) and unpaired tests. “+” indicates outlier.

Figure 7 (left) illustrates the parameters significantly affected by dye loading during ischemia and reperfusion. It is evident (see Figures 2, 7) that the character of changes is very similar in both groups. However, the differences between the non-stained and the stained hearts are based on the effect of the dye before the ischemia onset.

To assess the electrophysiological response of myocardium to ischemia regardless the dye-related changes, the same statistical approach was applied on Δ values of the parameters. Distributions of such corrected data are shown in Figure 7, right. Generally, the correction procedure led to diminishing (especially in the reperfusion) or, on the contrary, emphasizing (in the ischemia) of the differences between the two groups (compare the plots in Figure 7, left and right). Particularly, in QRS_D_, the combination of both phenomena was observed which resulted in elimination of the differences in the washout and the reperfusion on one hand and appearing significant difference in the middle of ischemia on the other hand. The latter seems to be due to significantly higher QRS prolongation developed in the non-stained hearts as compared to that in the stained ones. The differences presented in RR_D_ and QT_D_ through the whole time period were almost completely eliminated by the correction. The magnitude of the parameters’ changes relative to their non-ischemic level–namely increase of RR_D_ and decrease of QT_D_–was similar in the stained and the non-stained hearts.

In reperfusion, the parameters affected by the ischemia gradually returned to their non-ischemic levels. Generally, ischemia-related alterations of the parameters vanished during approx. the first 1–3 min of the reperfusion. In the stained hearts, the recovery was usually faster (by approx. 1 min) than in the non-stained hearts.

#### Detection of Ischemia Onset

**FIGURE 8:**
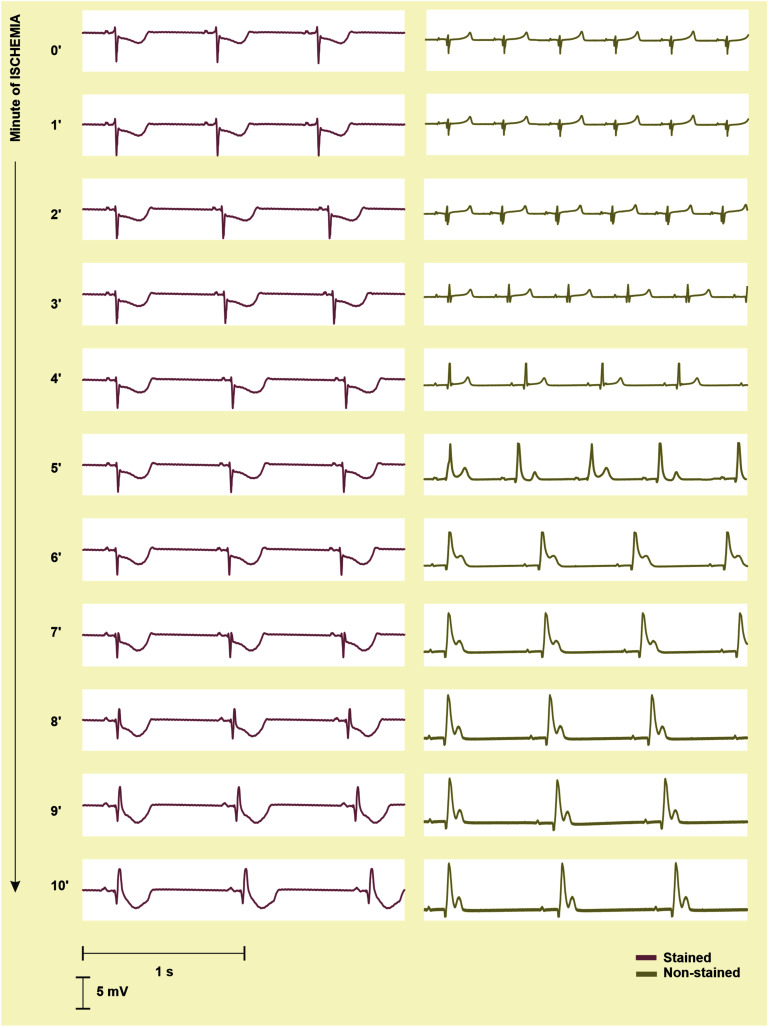
Electrograms (EG) recorded before the onset (0′) and during 10-min ischemia in the stained (*left*) and the non-stained (*right*) rabbit isolated heart. In the stained heart EG, ischemia manifestations (inverted, increased and widened QRS, elevated/depressed ST, inverted T wave) appear in the 8th–9th minute, whereas in non-stained heart, the first ischemia-induced changes are obvious in EG from the 2nd–3rd minute. As a result, morphology of EG recorded in the stained and the non-stained heart significantly differs at the beginning and middle of ischemia and become similar at the end of the ischemia.

To quantify the progress of ischemia in the stained and the non-stained hearts and to evaluate the success of EG-based ischemia detection, QRS_A_ and T_A_ were assessed statistically. As mentioned above, under non-ischemic conditions, no differences were found between the groups (see corresponding values at the end of the washout in Figure 7, left). Nevertheless, prominent ischemic changes (namely increase of the values from the 2nd–3rd minute of the ischemia) were indicated 3–4 min earlier in the non-stained hearts (according to the paired test relative to the end washout values, see Figure 7, left). It resulted in significant differences between the groups in the middle of ischemia, as can be clearly seen on the raw EGs from Figure 8. In case of QRS_A_, the significant difference between the groups remained even after data correction (compare Figure 7 left and right).

**FIGURE 9:**
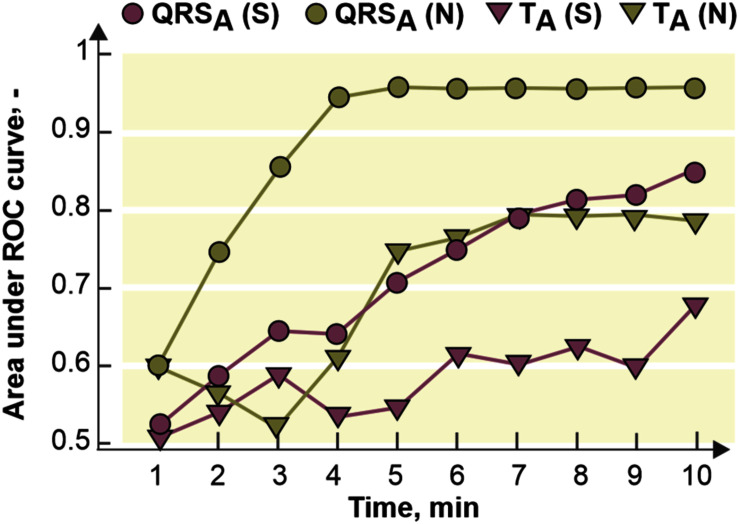
Area under receiver operating curve (ROC) for maximal absolute QRS deviation (QRS_A_) and maximal deviation of ST-T interval (T_A_) calculated for every minute of the ischemia. S, *N*, stained and non-stained hearts, respectively.

The fact that ischemic patterns (reflected in both depolarization and repolarization parameters) are less prominent and time-delayed in the stained hearts comparing to the non-stained ones (see Figure 7) leads to poorer performance of ischemia detection in these data. It is illustrated in Figure 9, where AUCROC values calculated for QRS_A_ and T_A_ in every minute of the ischemia are shown. Generally, both parameters discriminated between the non-ischemic and the ischemic values better in the non-stained hearts than in the stained ones. In both groups, QRS-based parameter had better discrimination ability than ST-T-based one. In the stained and the non-stained hearts, AUCROC of QRS_A_ reached the value higher than 0.8 (corresponds with a good discrimination ability) in the 8th and the 3rd minute of the ischemia, respectively. Only in the 10th minute of the ischemia, AUCROC calculated for the stained hearts reached similar value (approx. 0.87) as that calculated for the non-stained hearts in the 3rd minute of ischemia. In case of T_A_, AUCROC was close to 0.8 from the 7th minute of the ischemia in the non-stained hearts and did not reach this value in the stained hearts even at the end of the ischemic period.

## Discussion

### Direct Effects of Di-4-ANEPPS Under Non-ischemic Conditions

Di-4-ANEPPS is the most widely used VSD in isolated heart models despite the repeated reports of controversial effect on cardiac electrogram parameters. The abovementioned findings confirm alteration of these parameters caused by di-4-ANEPPS under both non-ischemic and ischemic conditions.

In accordance with the findings presented in this study, decreased spontaneous heart rate (or increased RR duration) and slowed impulse conduction through the ventricles have been reported in isolated hearts stained by di-4-ANEPPS in almost all commonly used laboratory animal species (Nygren et al., 2003; Novakova et al., 2008; Larsen et al., 2012). Decreased heart rate may be caused either by the effect of di-4-ANEPPS on ion channels in SA node or by impairment of coupling between the nodal cells. The SA node is a heterogeneous complex of nodal cells and other tissues (Shattock and Rosen, 2006). The heterogeneous ion channel expression in different parts of the node and the variation of ion channels within a single nodal cell make the experimental verification of above hypothesis very difficult. Nevertheless, the effect of di-4-ANEPPS seems to be very similar to the effect of specific ion channel blocker ivabradine. Ivabradine selectively inhibits an important potassium depolarization current (the “funny” current, I_f_) which controls the rate of spontaneous firing in SA node. As reported previously, administration of ivabradine decreases heart rate and reduces severity of ischemia (Ng et al., 2013). However, the direct effect of di-4-ANEPPS on I_f_ has not been studied yet. Future studies are needed to address this issue.

To our best knowledge, evaluation of cardiac impulse conduction throughout the atria and AV node (based on analysis of P_D_ and PQ_D_, respectively) in rabbit isolated hearts stained with di-4-ANEPPS was addressed for the first time in this study. Significant slowing of AV node conduction (prolonged PQ) was observed in the stained hearts. The effect was only transient during the dye loading and disappeared in the washout. For comparison, sustained prolonged PQ and concurrent transient AV block were observed in di-4-ANEPPS stained rat hearts (Nygren et al., 2003).

Significant irreversible widening of QRS complex was observed in rabbit isolated hearts during the dye loading. It is well known that drug-induced QRS prolongation is often associated with block of I_Na_ which results in slow depolarization and, consequently, reduced conduction velocity throughout the ventricles (Gintant et al., 2011).

As all the above results (significant decrease of heart rate, mild transient PQ prolongation and irreversible QRS widening) might be connected with I_Na_ inhibition, experiments testing the effect of di-4-ANEPPS on I_Na_ on differentiated NG108-15 cells were performed. The NG108-15 is a hybrid cell line originating from neuronal tissue. Significant inhibition of sodium current density was detected in the NG108-15 cells incubated in the presence of di-4-ANEPPS. DMSO, the solvent used for dye dilution in our experiments, did not contribute to observed current inhibition. Multiple isoforms of voltage gated sodium channels are expressed in NG108-15 cell line. Main channel isoform is the Na_V_1.7 channel (Kawaguchi et al., 2007; Liu et al., 2012). This isoform is present to a certain extent also in cardiac tissue as reported for a newborn rabbit sinoatrial node (Baruscotti et al., 1997), induced pluripotent stem cell-derived cardiomyocytes (Moreau et al., 2017) and in sinoatrial node of mouse heart (Marionneau et al., 2005). Further, it shares more than 85% amino acid sequence similarity with the Na_V_1.5, which is considered to be the principal cardiac sodium channel. Therefore, we may presume that di-4-ANEPPS inhibits I_Na_ also in cardiac tissue and may contribute to abovementioned changes in heart rate, PQ and QRS durations. A description of the effect of the dye on various sodium channel isoforms and/or under various conditions (e.g., various staining protocols, washout, and ischemia) might be addressed in future research.

Also, the decreased mean coronary flow detected during the staining has to be considered in the interpretation of the abovementioned results. Decreased coronary flow in isolated heart stained by di-4-ANEPPS has been previously reported in mouse, guinea pig, and rabbit model (Nygren et al., 2000; Novakova et al., 2008). Significantly decreased coronary flow might lead to ischemia and subsequently to a decrease in heart rate and conduction velocity. However, the coronary flow depends on numerous factors and the direct vasoconstriction effect of di-4-ANEPPS has not been conclusively confirmed yet. Also, no production of hydroxyl radicals in consequence of di-4-ANEPPS loading was reported (Novakova et al., 2008). It indicates that the decrease in coronary flow during the staining may not lead to ischemia. Moreover, coronary flow in isolated heart model is usually measured as an outflow of the perfusion solution from the heart or an overflow of the solution from the bath where the preparation is placed (as in our setup). Such measurement is somewhat rough and therefore coronary flow has to be interpreted only as subsidiary parameter for monitoring the overall physiological stability of the preparation. Future studies in an isolated coronary artery or any other eligible model are needed to describe vasoconstriction properties of di-4-ANEPPS.

Considering the absence of pathological episodes before the staining (i.e., in the stabilization), the presence of impaired rhythm in following periods may be presumed as the effect of the dye. The onset of junctional rhythm in the stained hearts might be induced by slowed conduction velocity and decreased heart rate. Above observations were accompanied by the changes in ST-T interval morphology, which represents the repolarization process in the heart. Therefore, it could be assumed that di-4-ANEPPS affects cardiac potassium channels, too. These alterations could be eliminated by the washout procedure of proper duration (at least 20 min, as was used in the present study).

Some of the results agree with observations in guinea pig isolated heart (QRS prolongation, slowing impulse propagation–Larsen et al., 2010, 2012) despite the different staining procedures used: bolus of highly concentrated dye acutely administered directly to the aorta–i.e., “fast” staining procedure–in case of guinea pig experiments vs. “slow” staining by perfusion with the low dye concentration used in the present study. Similar phenomena might be expected in rabbit hearts which underwent acute di-4-ANEPPS administration due to the similarity in the mechanism of AP propagation (in terms of depolarisation and repolarisation currents involved in the process) in guinea pig and rabbit myocardium (Varró et al., 1993). As regards RR and QT duration in rabbit EG, the changes appeared in the first minutes of the staining procedure and, thus, would likely be present in the experiments with acute dye application, too.

The data from the experiments with di-4-ANEPPS (regardless the staining procedure used) should be interpreted with caution and the obtained results should be validated using different experimental tools if possible. Particularly, the assessment of drug effects in isolated heart stained by di-4-ANEPPS might be complicated due to confounding effects of the dye, such as QT and QRS prolongation present from the beginning of the staining. However, the former (or corresponding QT_C_ shortening) could be explained by the response to RR alteration and, thus, should not be interpreted as a sign of direct arrhythmogenic effect. These aspects are of special impact in studies using rabbit heart for investigating the arrhythmogenic potential of certain drugs (Kaese et al., 2013).

The abovementioned findings are not the result of phototoxic effect of the dye, but the direct effects of di-4-ANEPPS on the rabbit myocardium. The heart tissue illumination (and, as a consequence, dye molecules excitation) was performed locally by six optical fibers with diameter of 200 μm (Kolarova et al., 2010). The excitation was induced after the washout period. Furthermore, the heart preparation was kept in the dark during the whole experiment to reduce phototoxic and to eliminate photobleaching effects of the dye.

An important strength of the study is that no excitation-contraction uncouplers was used. In most studies, uncouplers (such as 2,3-butanedione monoxime, cytochalasin D, and blebbistatin) are applied to reduce the motion artifact in AP records. Possible side effects of such agents may complicate the analysis of electrogram and may lead to incorrect interpretation of the results (Baker et al., 2004; Swift et al., 2012; Brack et al., 2013; Kappadan et al., 2020).

### The Effects of Di-4-ANEPPS in Ischemia and Reperfusion

This study revealed that ischemia manifestations in EG of rabbit isolated heart is affected by di-4-ANEPPS staining. Generally, the ischemia is manifested as similar changes (e.g., QRS prolongation and bradycardia) of EG parameters in both stained and non-stained hearts. Therefore, it can be concluded that rabbit isolated heart stained with this VSD is an appropriate tool for electrophysiological investigation of myocardial ischemia. However, prominent ischemic patterns appeared in the stained hearts with 3–4 min delay as compared to the non-stained ones.

First, significant QRS prolongation was observed in both groups. QRS duration reflects conduction of depolarisation through the ventricular myocardium. Under ischemia, conduction velocity is decreased due to impaired metabolism in myocardium, which is manifested in EG as QRS complex widening. Similar phenomenon associated with severe and global ischemia in human has been reported previously (Takaki et al., 1999). QRS prolongation can be partially explained by less steep R peak observed in EG recorded during the ischemia (not shown). This is in accordance with the results of AP analysis in rabbits isolated hearts, where increased upstroke duration and decreased amplitude of AP were observed in both global and regional ischemia protocols (Matiukas et al., 2009; Kolarova et al., 2010; Martišiene et al., 2015).

Second, sinus bradycardia was observed during ischemia in both groups which might be explained by impaired function of SA node. According to the results on isolated rabbit SA node, such a bradycardia seems to be due to reduced inward Na–Ca exchange current I_NCX_ and T-type Ca current I_Ca,T_ [verified on pacemaker cells perfused by “ischemic” solution-i.e., solution with omission of glucose, pH 6.6 and additional upgrades for evaluation the role of increased serum (K)] (Du and Nathan, 2007).

Third, shortened QT interval found in both experimental groups is the result of decreased AP duration, as previously reported in the same experimental setup with global ischemia (Kolarova et al., 2010) as well as in rabbit isolated heart with regional ischemia induced by LDA occlusion (Martišiene et al., 2015). Morphological parameters, such as QRS_A_ and T_A_, were significantly changed in the ischemia in both stained and non-stained hearts. It is in accordance with well-known ECG patterns associated with myocardial ischemia or infarction (Wagner et al., 2009).

Despite above obvious similarities in both experimental groups, the onset of significant ischemic changes in EG parameters in the stained hearts was generally delayed by 1–2 min as compared to the non-stained hearts. Moreover, the magnitude of the changes in the stained hearts was not as prominent as in the non-stained ones. Interestingly, such trend was observed also in parameters with no evident response to di-4-ANEPPS under non-ischemic conditions. Attenuated ischemia progress in the stained hearts may be explained by decreased resting heart rate. Despite the fact that the character of ischemic changes of RR interval duration was the same in the stained and the non-stained hearts (as confirmed by ΔRR_D_ values), the baseline value of RR_D_ (i.e., immediately before perfusion stopping) in the stained hearts was shifted to the higher values as a response to the dye. As a consequence, heart rate observed in the stained hearts immediately before as well as in the ischemia and the reperfusion was generally lower than that of the non-stained hearts. It has been previously reported that slowing heart rate significantly reduces cardiac energy consumption and therefore may reduce the severity of ischemia and enhance recovery in reperfusion (Bernier et al., 1989; Ceconi et al., 2009; Ng et al., 2013). Furthermore, in patients treated with beta or calcium blockers, which reduce heart rate and systolic arterial blood pressure, false-negative response to exercise test may be obtained due to decreased myocardial oxygen requirements (Surawicz and Tavel, 2008).

Reduced severity of myocardial ischemia in the stained hearts is reflected on EG as attenuated ischemic patterns. It further leads to late and inaccurate ischemia detection, even when QRS_A_ and T_A_ are used (Ronzhina et al., 2017). In the control group, both parameters seem to be appropriate for discrimination between the non-ischemic state and earlier (namely, the 3rd minute of ischemia in case of QRS_A_) or later (namely, the 7th minute of ischemia in case of T_A_) phase of the ischemia. On the other hand, even in the non-stained hearts, none of EG parameters was able to detect successfully first 2 min of ischemia. This might be caused by low sensitivity of the parameters or EG itself to the earliest phase of ischemia. Another explanation of this phenomenon could be the residual oxygen present in the myocardium of the non-working heart (i.e., heart not pumping against the load) in the amount sufficient to supply the tissues for a limited time. As a result, the onset of substantial oxygen deficit in myocardium (leading to evident alteration of cardiac electrical activity and, thus, manifested in EG) does not appear immediately, but a few minutes after the perfusion is stopped. Although this hypothesis is difficult to verify, it is in accordance with other studies on rabbit isolated hearts, where the first pronounced changes in the heart electrical activity were found 3–5 min after perfusion stopping and 30–90 min long ischemic period was needed to induce myocardial infarction (Bernardo et al., 1999; Feng et al., 2013; Martišiene et al., 2015).

Above findings and their consequences should be considered when designing experimental protocol with di-4-ANEPPS and analyzing and interpreting obtained data. Particularly, delayed and probably less pronounced response to ischemia should be expected in the hearts stained with the dye. If ischemia, ischemia-induced arrhythmias or myocardial infarction are under investigation, the prolongation of ischemic period (i.e., experimental period with perfusion stopped) and/or increase of heart rate by pacing may be considered in the stained hearts to achieve appropriate results comparable with those observed in the non-stained hearts. Generally, special care should be taken if the results from experiments with di-4-ANEPPS are compared with those from control group without dye administration. In such case, possible irreversible changes of electrophysiological parameters appearing during staining or washout should be identified. Appropriate methodological correction (such as analysis of Δ values as proposed in this study) should be performed to avoid wrong interpretation and conclusions. Another way to avoid possible discrepancies in the results is use of stained hearts in both investigated and control groups.

Above results were obtained in rabbit isolated heart. However, the effects of di-4-ANEPPS may be less (or more) pronounced in the hearts of other species. It should be also noted that mentioned observations are valid only for di-4-ANEPPS and may not be simply applied on other VSDs.

## Conclusion

The results of the study provide novel valuable information for designing and interpreting electrophysiological measurements on rabbit isolated hearts under control and ischemic conditions in the presence of di-4-ANEPPS. It was demonstrated that common voltage-sensitive dye di-4-ANEPPS only slightly modulates impulse conduction through atria and AV node in rabbit isolated heart. On the other hand, di-4-ANEPPS significantly affects spontaneous heart rate and ventricular conduction in rabbit isolated heart. It was shown that di-4-ANEPPS significantly inhibits tetrodotoxin-sensitive sodium current. Irreversible character of these changes results in altered response of stained hearts to ischemia. Particularly, delayed onset and less pronounced severity of ischemia were observed as compared to the non-stained hearts. Although no significant differences in mean number of VPBs were found between the stained and the non-stained hearts in ischemia as well as in reperfusion (see Figure 6B), all abovementioned results indicate increased arrhythmogenicity.

Thus, our data strongly suggest that suitable control group, duration of washout (in all experiments with the dye) and ischemic period (in experiments with ischemia), method for electrogram evaluation, and other methodological issues should be carefully considered in experiments with VSD di-4-ANEPPS. Such approach can ensure higher quality and reliability of experimental investigation of various phenomena, such as myocardial ischemia and drug effects.

## Data Availability Statement

The raw data supporting the conclusions of this article will be made available by the authors, without undue reservation.

## Ethics Statement

The animal study was reviewed and approved by The animal experiments were carried out with respect to the recommendations of the European Community Guide for the Care and Use of Laboratory Animals and followed the experimental protocols approved by the Committee for Ensuring the Welfare of Experimental Animals, Faculty of Medicine, Masaryk University.

## Author Contributions

MN, IP, JK, KF, and MR designed the experimental protocol for rabbit isolated heart experiments. KF, TS, and MN supplied the animals. KF, TS, MR, OJ, JK, IP, and MN conducted the experiments on rabbit isolated hearts. MR, LM, RS, OJ, TS, and JK contributed to processing and analysis of data from isolated rabbit heart. LL, KO, and MP designed the experimental protocol for cell experiments, conducted the experiments, and processed and analyzed path-clamp data. MR, TS, LL, and MN wrote the manuscript. JK and IP contributed to critical reading of the manuscript and reviewed the literature. All authors contributed to the article and approved the submitted version.

## Conflict of Interest

The authors declare that the research was conducted in the absence of any commercial or financial relationships that could be construed as a potential conflict of interest.
